# On the Fourth *Diadema* Species (*Diadema*-sp) from Japan

**DOI:** 10.1371/journal.pone.0102376

**Published:** 2014-07-23

**Authors:** Seinen Chow, Yoshikazu Kajigaya, Hiroaki Kurogi, Kentaro Niwa, Takuro Shibuno, Atsushi Nanami, Setuo Kiyomoto

**Affiliations:** 1 National Research Institute of Fisheries Science, Yokohama, Kanagawa, Japan; 2 Yokosuka Laboratory, National Research Institute of Aquaculture, Yokosuka, Kanagawa, Japan; 3 Ishigaki Laboratory, Seikai National Fisheries Research Institute, Ishigaki, Okinawa, Japan; 4 Seikai National Fisheries Research Institute, Nagasaki, Nagasaki, Japan; Chang Gung University, Taiwan

## Abstract

Four long-spined sea urchin species in the genus *Diadema* are known to occur around the Japanese Archipelago. Three species (*D. savignyi*, *D. setosum*, and *D. paucispinum*) are widely distributed in the Indo-Pacific Ocean. The fourth species was detected by DNA analysis among samples originally collected as *D. savignyi* or *D. setosum* in Japan and the Marshall Islands and tentatively designated as *Diadema* -sp, remaining an undescribed species. We analyzed nucleotide sequences of the cytochrome oxidase I (COI) gene in the “*D. savignyi*-like” samples, and found all 17 individuals collected in the mainland of Japan (Sagami Bay and Kyushu) to be *Diadema*-sp, but all nine in the Ryukyu Archipelago (Okinawa and Ishigaki Islands) to be *D. savignyi*, with large nucleotide sequence difference between them (11.0%±1.7 SE). *Diadema*-sp and *D. savignyi* shared Y-shaped blue lines of iridophores along the interambulacrals, but individuals of *Diadema*-sp typically exhibited a conspicuous white streak at the fork of the Y-shaped blue iridophore lines, while this feature was absent in *D. savignyi*. Also, the central axis of the Y-shaped blue lines of iridophores was approximately twice as long as the V-component in *D. savignyi* whereas it was of similar length in *Diadema*-sp. Two parallel lines were observed to constitute the central axis of the Y-shaped blue lines in both species, but these were considerably narrower in *Diadema*-sp. Despite marked morphological and genetic differences, it appears that *Diadema*-sp has been mis-identified as *D. savignyi* for more than half a century.

## Introduction

Long-spined sea urchins in the genus *Diadema* Gray, 1825 are widespread species found in tropical and sub-tropical regions [1], [2]. Four *Diadema* species are reported from the southern part of the Japanese Archipelago [3]. *D. savignyi* (Audouin, 1829) and *D. setosum* (Leske, 1778) are the most abundant and well known, both having the widest geographic range in the Indo-Pacific Ocean of any species in the genus. The Hawaiian Islands and South Pacific [4] are considered the core distribution for *D. paucispinum* A. Agassiz, 1864, and the species is suspected to inhabit other parts of the tropical Indo-Pacific [1], and has been observed in Japanese waters [3]. Using mtDNA analysis, the fourth species of *Diadema* was found among samples collected from Japanese and Marshall Island samples initially identified as *D. savignyi* or *D. setosum* and tentatively designated as *Diadema*-sp [3]. Unfortunately, the specimens used for the genetic analysis were not also subjected to morphological analyses. Since first being reported in 2001 *Diadema*-sp has received almost no attention from researchers [3].

We have undertaken monthly sampling of *Diadema* species from the Arasaki area of Sagami Bay, Japan, in order to investigate species abundance and the annual reproductive cycle, in which two types of *Diadema* morphologically corresponding to *D. savignyi* and *D. setosum* were observed. However, preliminary nucleotide sequence analysis of mtDNA cytochrome oxidase I gene (COI) from these samples indicated that our “*D. savignyi*” collected in Sagami Bay was the previously reported *Diadema*-sp [3]. Consequently, in the present study, we applied the COI gene and morphological analyses to elucidate the presence of *Diadema*-sp, among samples originally identified as *D. savignyi* collected in waters of mainland and the southern islands of Japan.

## Materials and Methods

### Ethics statement

All field surveys were approved by Nagai-machi Fishermen's Cooperative Association (Yokosuka, Japan), Gonoura-cho Fishermen's Cooperative Association (Nagasaki, Japan), Yaeyama Fishermen's Cooperative Association (Ishigaki, Japan), and Sesoko Station of the University of the Ryukyus (Okinawa, Japan). The species of the genus *Diadema* sampled in the present study were not endangered in Japan and verbal permits for collecting *Diadema* species were obtained from the above organizations.

### Sampling

**Figure 1:**
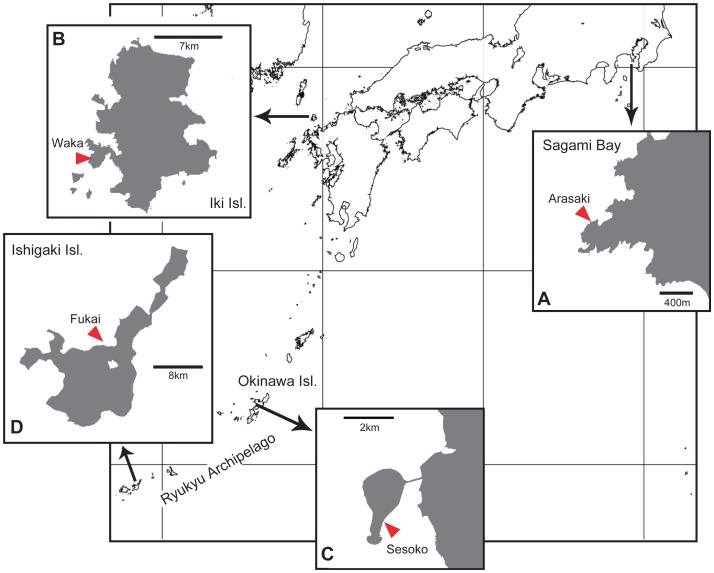
Collection localities for *Diadema* samples in Japan. A, Arasaki, Sagami Bay; B, Waka, Iki Island; C, Sesoko, Okinawa Island; D, Fukai, Ishigaki Island.

Individuals of “*D. savignyi*-like” and *D. setosum* were sampled from four locations between December 2011 and May 2013 (Figure 1, Table 1). Only urchins with a test diameter of 25 to 60 mm were sampled. “*D. savignyi*-like” and *D. setosum* individuals were easily identified underwater. *D. setosum* was characterized by a bright colored periproctal cone (anal tube) with a conspicuous orange (or red) ring, and five conspicuous white spots on the median naked interambulacral areas. In comparison, “*D. savignyi*-like” individuals did not typically project a periproctal cone, and when a cone was observed, a prominent orange ring was not present. In addition, “*D. savignyi*-like” individuals exhibited five blue lines of iridophores along the interambulacrals, and a pentamerous ring around the periproctal region. All individuals were photographed underwater *in situ* or alive in aquaria following collection and transfer to the laboratory.

**Table 1:** *Diadema* sample collection data for mtDNA COI analyses.

Morphotype	code	Date collected	N	Locality (see Fig. 1)
“*Diadema savignyi*”	DSV1-8	December 2011	8	Arasaki, Sagami Bay (A)
	DSV9-17	February 2012	9	Waka, Iki Island (B)
	DSV18-19	May 2013	2	Sesoko, Okinawa Island (C)
	DSV20-26	October 2013	7	Fukai, Ishigaki Island (D)
*Diadema setosum*	DST1-8	December 2011	8	Arasaki, Sagami Bay (A)
	DST9-10	May 2013	2	Sesoko, Okinawa Island (C)

### DNA extraction, amplification and sequencing

Tube feet or muscle tissue surrounding the Aristotle's lantern was collected from each individual for genomic DNA extraction. The remaining body was immersed in bleach, primarily consisting of sodium hypochlorite to remove spines and other soft tissues to examine test morphology. Primer sequences used for PCR and nucleotide sequencing were as follows: COI120F (5′TTCTTCATGGTAATGCCAAT 3′) and COI1300R (5′ATTCCGGCTARACCTAAGAA3′). PCR was performed in a 20 µl reaction mixture containing 2 µl 10x buffer, 1 mM each dNTPs, 0.4 µM each primer, 0.8 units EX Taq polymerase (Takara, Japan), and DNA template. The reaction mixtures were preheated at 94°C for 4 min, followed by 30 amplification cycles (94°C for 30 sec, 50°C for 30 sec, and 72°C for 50 sec), with a final extension at 72°C for 7 min. PCR products were treated with ExoSAP-IT (Amersham Biosciences) to remove primers. Sequences were generated on an automated sequencer (ABI Prism 310; Applied Biosystems, Foster City, CA, USA) using the ABI Big-dye Ready Reaction kit (Applied Biosystems) following standard cycle sequencing protocol.

### Alignment and sequence analyses

Sequence chromatograms were manually checked using ChromasPro v1.42 (Technelysium Pty Ltd), and aligned by MEGA v6 [5]. *D. setosum*-a (AY012747 and AY012746), *D. setosum*-b (AY012732 and AY012733), *D. savignyi* (AY012742 and AY012743), and *Diadema*-sp (AY012744 and AY012745) sequences previously reported [3] were incorporated into the phylogenetic tree construction. Neighbor-joining (NJ) and maximum likelihood (ML) methods were applied to reconstruct phylogenetic trees using MEGA v6 [5], in which the gamma-corrected Kimura's two parameter (K2P) distance was selected as the best-fit model for nucleotide substitution.

### Morphological analyses

Underwater photographic images of live individuals were compared. Diameter and height of naked tests were measured, and primary tubercle arrangements on the interambulacral coronal plates were recorded.

## Results

### Genetic analyses

**Figure 2:**
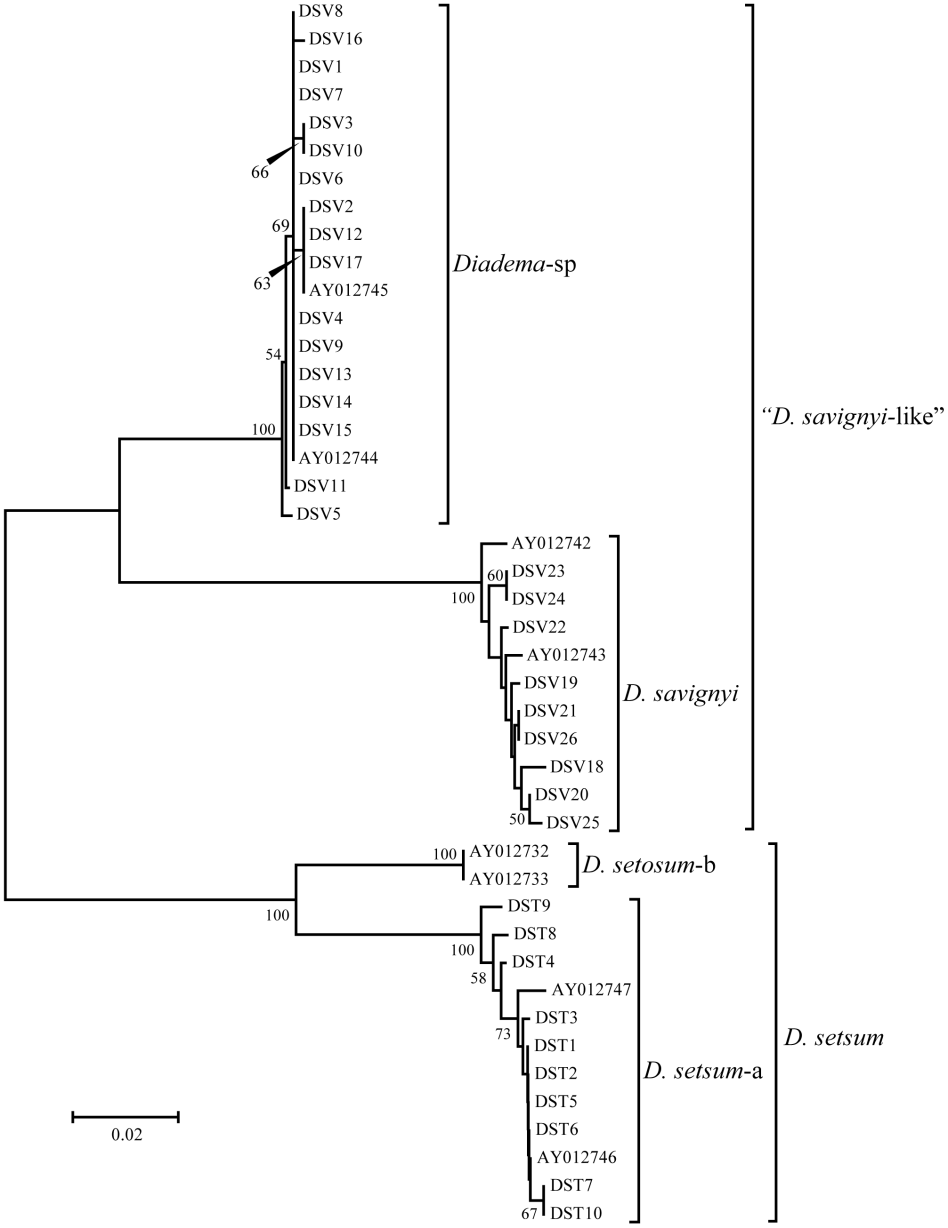
Neighbor-joining phylogenetic tree from COI sequence data. Bootstrap support (>50%) after 1,000 replications is shown at each node. See Table 1 for species designations.

Of the 758 to 984 nucleotide sequences resolved in 36 individuals, a 526 bp region was shared with previously reported sequence data [3] and used to reconstruct phylogenetic trees. All sequences obtained in this study are available in DNA Data Base of Japan (DDBJ) (AB909922–AB909957). NJ and ML trees represented essentially the same tree topology. Two major clades corresponding to “*D. savignyi*-like” and *D. setosum* were observed, each of which was further divided into two sub-clades (Figure 2). All clades were supported by high bootstrap values. All eight “*D. savignyi*-like” individuals (DSV1-8) collected in Sagami Bay, and nine (DSV9-17) in Iki Island (Figure 1A, B) were clustered in the *Diadema*-sp sub-clade, with small nucleotide sequence divergence among individuals (K2P: 0.2%±0.1 SE). All nine “*D. savignyi*-like” individuals (DSV18-26) collected in the Ryukyu Archipelago (Figure 1C, D) were clustered in the *D. savignyi* sub-clade, and exhibited small nucleotide divergence among individuals (0.6%±0.2 SE). On the other hand, average sequence divergence between *Diadema*-sp and *D. savignyi* was much larger (11.0%±1.7 SE). All 10 individuals of our *D. setosum* (DST1-10) were clustered in the *D. setosum*-a sub-clade, and nucleotide sequence divergence among individuals was small (0.5%±0.2 SE), while the divergence between *D. setosum*-a and *D. setosum*-b was 7.3%±1.3 SE. The largest divergence (13.9–18.7%) was observed among individuals representing *D. setosum* and “*D. savignyi*-like” clades.

### Morphological observations

**Figure 3:**
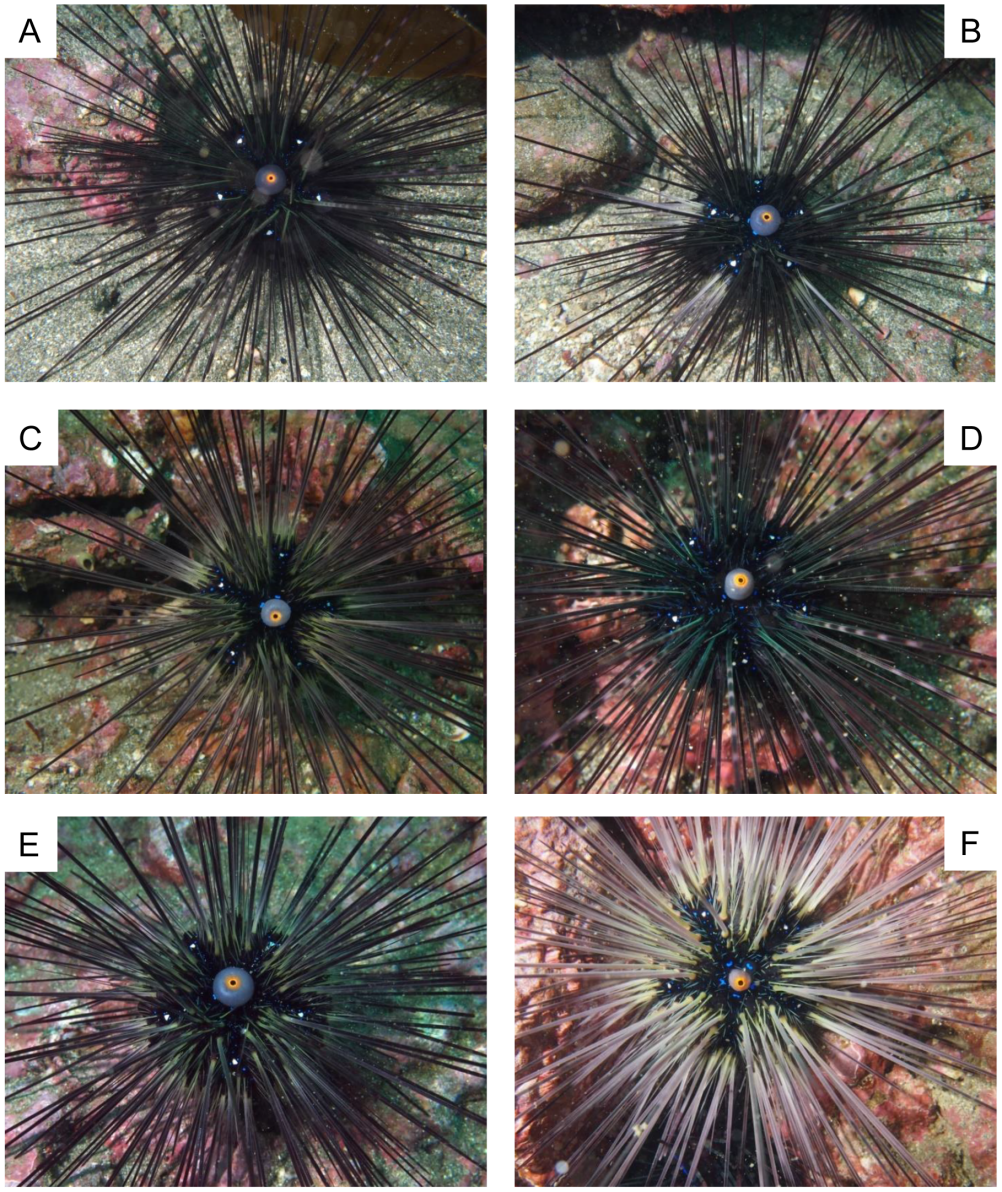
Underwater images of *Diadema setosum*. A to F correspond to DST1 to 6 (see Table 1). Note conspicuous white spots on the median naked interambulacral areas, and light colored periproctal cone with orange or red ring.

**Figure 4:**
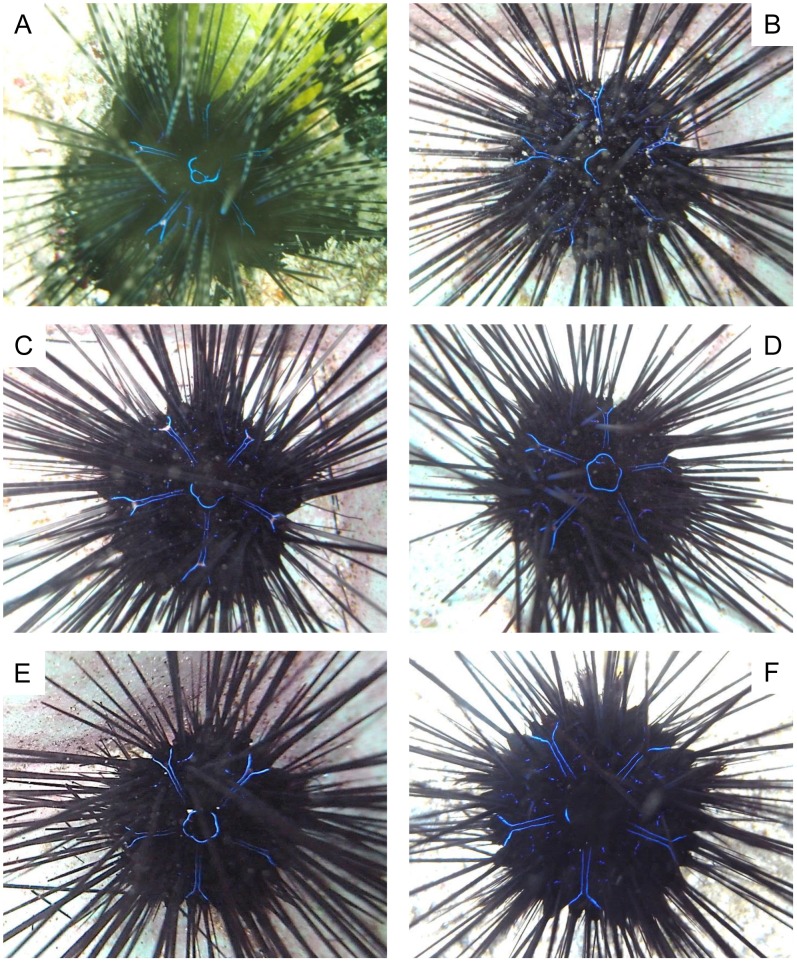
Underwater images of *Diadema savignyi*. A to F correspond to DSV19, 21, 23 to 26 (see Table 1). Note the central axis of the Y-shaped blue lines of iridophores along the median naked interambulacral area was approximately twice as long as the V-component, and comprised of two parallel lines.

**Figure 5:**
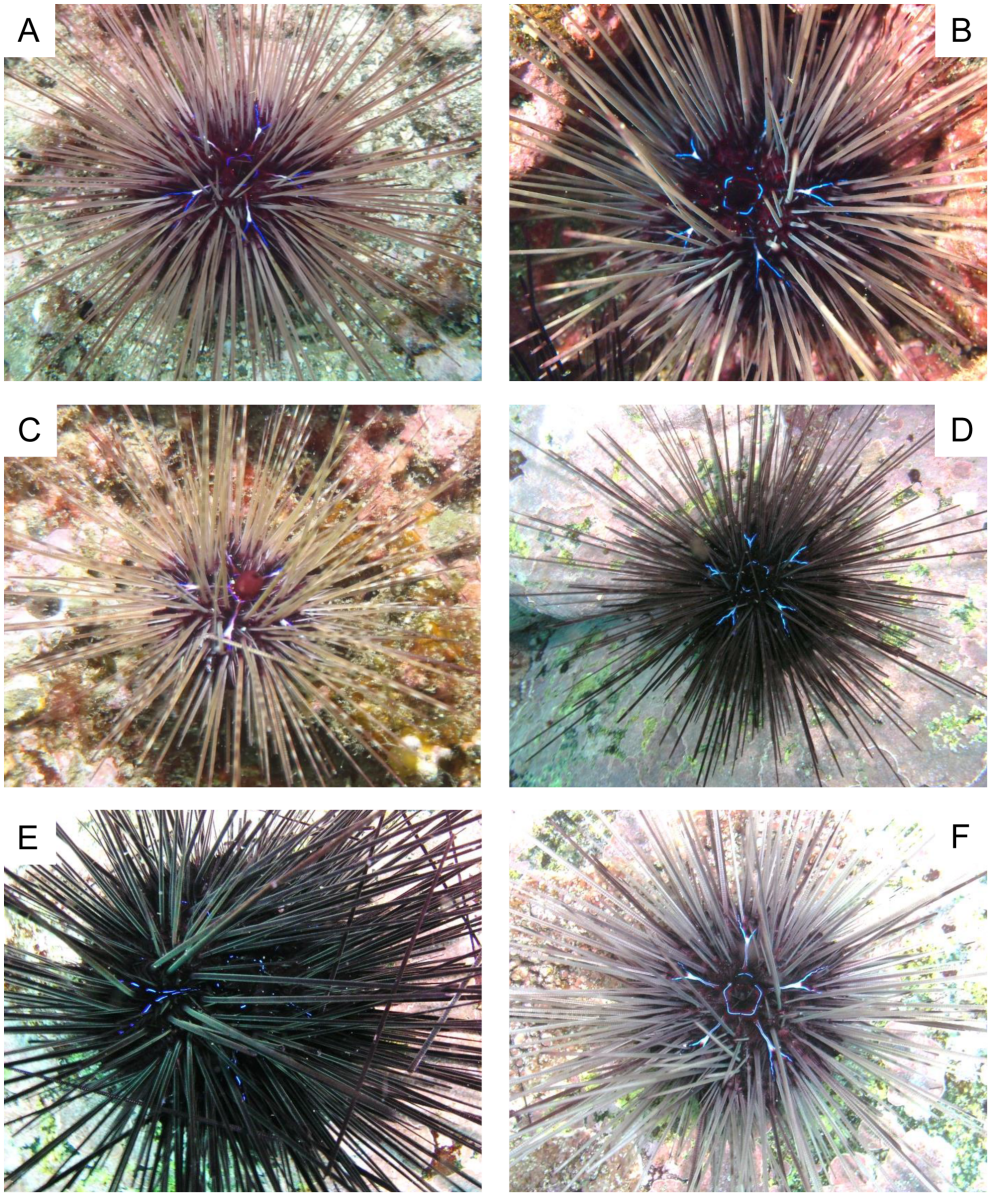
Underwater images of *Diadema*-sp. A to F correspond to DSV1 to 3, and 12 to 14 (see Table 1). Note the central axis of the Y-shaped blue lines of iridophores along the median naked interambulacral area which was represented by a single line, and similar in length to the V-component. Projection of periproctal cone was observed in one individual (C), but brown in color and an orange ring was not observed. The Y-shaped blue lines of iridophore were usually accompanied by a conspicuous white streak at the fork region (A to C, F), while individuals exhibiting a Y-shaped blue broken line (E) and the absence of a white streak (D, E) were rarely observed.

The species designations used below were derived from mtDNA COI sequence analysis results in the present study. Spine coloration and banding patterns showed notable variability in all species (Figure 3 - *D. setosum*, Figure 4 - *D. savignyi*, Figure 5 - *Diadema*-sp). Compared to *D. setosum*, *D. savignyi* and *Diadema*-sp lacked conspicuous white spots on the interambulacral area, and the periproctal cone projection was also usually absent. Even when the periproctal cone was observed, it was dark in color and lacked an orange ring (Figure 5C). Furthermore, a blue pentamerous ring of iridophore was typically observed around the periproct. Five Y-shaped blue lines of iridophores that intersect perpendicular to the pentamerous ring were present, with the Y located on the median naked interambulacral area in both *D. savignyi* and *Diadema*-sp. Notable differences between *D. savignyi* and *Diadema*-sp were observed in the Y-shaped blue lines. In *Diadema*-sp, the line was usually accompanied by a conspicuous white streak at the fork region (Figure 5A–C, F), which was absent in *D. savignyi*. In *D. savignyi*, the central axis of the Y-shaped blue line was approximately twice as long as the V-component, and comprised of two parallel lines (Figure 4). The central axis of the Y-shaped blue line in *Diadema*-sp was comparable in length to the V-component, and consisted of two much narrower parallel lines that without close examination appeared like a single line (Figure 5). *Diadema*-sp individuals, with a Y-shaped blue broken line (Figure 5E) and no prominent white streak (Figure 5D, E) were observed, but rarely. The blue lines that were present in *D. savignyi* and *Diadema*-sp when observed underwater became faint once the individuals were examined in air and became invisible after fixation.

**Figure 6:**
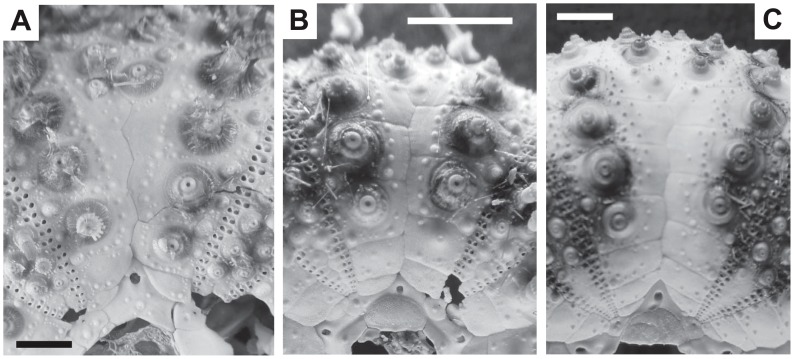
Primary tubercle arrangements on the interambulacral areas (bar  = 5 mm). A, *Diadema*-sp.; B, *Diadema savignyi*; C, *Diadema setosum*. Outer and inner series of primary tubercles in *Diadema*-sp. were initiated on the 3^rd^ (rarely 2^nd^ or 4^th^) and 6^th^ (rarely 5^th^) coronal plates, respectively (A). Those in *D. savignyi* began on the 3^rd^ (rarely 2^nd^ or 4^th^) and 6^th^ (B); and in *D. setosum*, on the 3^rd^ or 4^th^ (rarely 5^th^) and 7^th^ or 8^th^ (rarely 6^th^) coronal plates (C).

*Diadema* tests were fragile, therefore many individuals used for genetic analyses were unfortunately destroyed in early attempts using 100% bleach. Consequently, additional *Diadema*-sp and *D. setosum* specimens were collected from the Arasaki area in January 2014, and these individuals, including seven *D. savignyi* (DSV20-26) from Ishigaki Island were immersed in 50% bleach for several hours with occasional gentle shaking. Naked test proportion (height/diameter) results were as follows: *D. savignyi* (n = 7), 0.482±0.032 SD; *Diadema*-sp (n = 29), 0.479±0.027 SD; and *D. setosum* (n = 20), 0.508±0.035 SD. A slight but significant difference was observed between *D. setosum* and *Diadema*-sp in the naked test proportion (height/diameter) among the three species examined (Kruskal-Wallis test, *P* = 0.021), but the proportion appeared not to be diagnostic. The apical system did not always remain in the original position, even after short bleach treatments, therefore the following sample sizes were measured for primary tubercle arrangements in naked tests; four of seven *D. savignyi* collected at Ishigaki Island, 19 of 29 *Diadema*-sp, and 17 of 20 *D. setosum* additionally collected in the Arasaki area. From these representative images of primary tubercles on the interambulacral area for each species were prepared (Figure 6). The following qualitative assessments were made for each species. In *Diadema*-sp, the outer series of primary tubercles was typically initiated on the 3^rd^ (rarely 2^nd^ or 4^th^) coronal plate, and the inner series on the 6^th^ (rarely 5^th^) plate (Figure 6A). In *D. savignyi*, the outer series began on the 3^rd^ (rarely 2^nd^ or 4^th^) coronal plate, and inner series on the 6^th^ plate (Figure 6B). In *D. setosum*, the outer series initiated on the 3^rd^ or 4^th^ (rarely 5^th^) coronal plates, and inner series on the 7^th^ or 8^th^ (rarely 6^th^) (Figure 6C). Therefore, the arrangement of interambulacral primary tubercles in naked tests was a poor diagnostic character between *D. savignyi* and *Diadema*-sp, while that of inner series may barely discriminate *D. setosum* from the other two species.

## Discussion

The present study is the first to report morphological attributes characterizing the as yet undescribed *Diadema*-sp. A previous phylogeographic study that examined population structure and speciation in *Diadema* species throughout global tropical regions indicated support for a new *Diadema* species [3]. Prior to this earlier widespread tropical phylogeographic study [3], only two *Diadema* species (*D. savignyi* and *D. setosum*) had been reported in Japanese waters [6], [7]. External morphological traits had beentypically used in diagnostic keys for *D. savignyi* and included the absence of the orange ring on the periproctal cone and blue lines of iridophores on the apical system and interambulacral midline [4], [7]. *Diadema*-sp, however, cannot be distinguished from *D. savignyi* based on these morphological traits. In a survey of sea urchins from Sagami Bay, Shigei [7] provided underwater photographs of “*D. savignyi*”, which resembled *Diadema*-sp based on prominent white streaks and a short central axis of the Y-shaped blue lines of iridophores. *Diadema* species observed in the Oki Islands, Sea of Japan, were reported as *D. savignyi* [8], but the published photograph shows traits characteristic of *Diadema*-sp. “*D. savignyi*” photographs provided in several Japanese guide books [9]–[11] also represented attributes of *Diadema*-sp. Furthermore, allozyme electrophoresis patterns were compared between *D. savignyi* and *D. setosum* collected in mainland of Japan, and allelic differences were observed between the two species at *G6P* and *AAT* loci [12]. However, Okinawa Island populations of *D. savignyi* and *D. setosum* shared the same alleles at these loci [13], [14]. Therefore, it is likely that “*D. savignyi*” collected from the mainland of Japan and used for allozyme analyses [12] may be *Diadema*-sp. Our mtDNA COI results revealed that the “*D. savignyi*-like” specimens collected in Sagami Bay and Iki Island were *Diadema*-sp, but those collected in the Ryukyu Archipelago were *D. savignyi*. A previous study indicated an greater number of *Diadema*-sp versus *D. savignyi* occurred along the coast of mainland of Japan, while *Diadema*-sp were not observed in the Okinawa Island area [3]. Therefore, the geographic range of *Diadema*-sp appears to be within mainland waters of Japan, and the taxon has been mis-identified as *D. savignyi* for more than half a century. *D. savignyi* appears to be much less abundant in mainland Japanese waters and the distribution appears to be restricted in the southern islands. *Diadema*-sp has only been reported from mainland Japan and Marshall Island region to date [3]. However, photograph images of “*D. savignyi*” taken in Australia (http://www.scuba-equipment-usa.com/marine/OCT04/Tropical_Spined_Sea_Urchin(Diadema_savignyi).html and http://bie.ala.org.au/species/urn:lsid:biodiversity.org.au:afd.taxon:386810d3-25a5-4952-be0a-f396fe5cab50) and Singapore (http://www.flickr.com/photos/wildsingapore/2440065733/) represent characteristics of *Diadema*-sp, suggesting that the species may have much wider distribution and has been mis-identified as *D. savignyi* in many occasions.

In the early 20th century, Clark [15], [16] reported distinctive *Diadema* individuals from Sagami Bay, and suggested that the populations might represent an undescribed species. Following several mainland Japan collections, Ikeda [17] suggested his specimens to be identical to Clark's, and described a new species *Diadema clarki* after Clark [15], [16]. *D. clarki* was supposed to be a synonym of *D. setosum* [6], [18], while Lessios et al. [3] cautiously suggested that the *Diadema*-sp might be one of Clark's undescribed *Diadema* species [15], [16] or *D. clarki* by Ikeda [17] based on the similar geographic origin. However, Clark's undescribed *Diadema* specimen exhibited only eight or nine coronal plates in each column [16], which markedly differs from all *Diadema* species examined in the present study. On the other hand, qualitative affinities were observed between *Diadema*-sp and *D. clarki*. Ikeda [17] reported the following in *D. clarki*; the inner series of interambulacral primary tubercles began on the 5^th^ or 6^th^ coronal plates, and conspicuous white streaks were present in the median naked interambulacral area instead of white spots observed in *D. setosum*. Although Ikeda [17] did not provide a written description, his photograph of a naked test of *D. clarki* showed that the outer series of the interambulacral primary tubercles begun on the 2^nd^ or 3^rd^ coronal plates. However, Ikeda [17] also reported the end of the periproctal cone in *D. clarki* to be orange as in *D. setosum*, suggesting that *Diadema*-sp is unlikely to be *D. clarki*. Consequently, the relationship between *Diadema*-sp and *D. clarki* is equivocal, so that further investigation on the distribution and intraspecific morphological variation in *Diadema*-sp is required prior to formal species description.
